# Influence of Levosimendan Postconditioning on Apoptosis of Rat Lung Cells in a Model of Ischemia–Reperfusion Injury

**DOI:** 10.1371/journal.pone.0114963

**Published:** 2015-01-21

**Authors:** Chengxin Zhang, Zhixiang Guo, Haiyuan Liu, Yinglu Shi, Shenglin Ge

**Affiliations:** 1 Cardiovascular Surgery Department, the First Affiliated Hospital of Anhui Medical University, Hefei, Anhui, China; 2 Oncology Department, The He Fei Hospital affiliated with An Hui Medical University, Hefei, Anhui, China; 3 Department of Cerebral Surgery, The Chest Hospital of Anhui province, Hefei, Anhui, China; University of Missouri, UNITED STATES

## Abstract

**Objective:**

To ascertain if levosimendan postconditioning can alleviate lung ischemia–reperfusion injury (LIRI) in rats.

**Method:**

One hundred rats were divided into five groups: Sham (sham), ischemia–reperfusion group (I/R group), ischemic postconditioning (IPO group), levosimendan postconditioning (Levo group) and combination postconditioning group of levosimendan and 5-Hydroxydecanoic acid (Levo+5-HD group). The apoptotic index (AI) of lung tissue cells was determined using the terminal deoxynucleotidyl transferase dUTP nick end labeling (TUNEL) assay. Expression of active cysteine aspartate specific protease-3 ( active caspase-3), Bcl-2 and Bax in lung tissue was determined by immunohistochemical staining. The morphopathology of lung tissue was observed using light and electron microscopy.

**Results:**

AI values and expression of active caspase-3, Bcl-2 and Bax of lung tissue in I/R and Levo+5-HD groups were significantly higher than those in the sham group ( P<0.05). AI values and expression of active caspase-3 and Bax were significantly lower, whereas that of Bcl-2 was higher significantly in the Levo group, compared with I/R and Levo+5-HD groups (P<0.05). Significant differences were not observed in comparisons between I/R and Levo+5-HD groups as well as IPO and Levo groups.

**Conclusion:**

LIRI can be alleviated by levosimendan, which simulates an IPO protective function. A postulated lung-protective mechanism of action could involve opening of mitochondrial adenosine triphosphate-sensitive potassium channels, relieving Ca2+ overload, upregulation of expression of Bcl-2, and downregulation of expression of active caspase-3 and Bax.

## Introduction

Acute lung injury (ALI) is caused by ischemia–reperfusion during cardiopulmonary bypass (CPB). Despite improvements in CPB, ALI remains the main cause of increasing postoperative mortality and increased duration of hospitalization [1, 2]. Prevention and treatment of lung ischemia–reperfusion injury (LIRI) are important subjects in clinical and basic scientific research [2–6].

Recently, ischemic postconditioning (IPO) has been suggested to be a new method to protect organs from ischemia–reperfusion injury [7–9]. Recent studies [9, 10] have shown that one of the main protective mechanisms of IPO is to activate mitochondrial adenosine triphosphate (ATP)-sensitive potassium (mitoK_ATP_) channels. Some studies have reported that specific openers of mitoK_ATP_ channels protect skeletal muscle and the liver from ischemia–reperfusion injury through alleviation of apoptosis [11, 12]. However, there are few reports focusing on the protective function of IPO in LIRI.

Levosimendan is a new positive inotropic drug. It increases the sensitivity between Ca^2+^ and myofilaments by combining with myocardial troponin C (cTnC) without increasing the intracellular Ca^2+^ concentration, i.e., [Ca^2+^]_i_, to strengthen myocardial contraction without increasing oxygen consumption in the myocardium. Simultaneously, levosimendan can open K_ATP_ channels to protect the myocardium by preventing calcium overload, stabilizing the structure of the cell membrane and mitochondrial membrane, and decreasing the production of oxygen free radicals [13–16]. Levosimendan also helps mitoK_ATP_ channels to decrease ischemia–reperfusion injury in the myocardium [15, 16]. We hypothesized that levosimendan postconditioning could have a protective role in LIRI because of apoptosis (an important factor causing lung-tissue injury during LIRI) [17]. We explored this mechanism using levosimendan postconditioning to tackle lung ischemia and observed its influence on apoptosis lung cells during LIRI in rats.

## Materials and Methods

### Ethical approval of the study protocol

The study protocol was approved by Animal Ethics Committee of Anhui Medical University (Hefei, China). Experimental procedures were carried out under the supervision of the Ethics Committee to minimize the suffering of animals.

### Establishment of the animal model

Experimental animals were provided by the Animal Laboratory Center of Anhui Medical University: 100 specific pathogen-free male rats (290–320 g). Rats were divided randomly into five groups. In the sham group, the hilum of the left lung was exposed after thoracotomy without blockage. In the ischemia–reperfusion (I/R) group, the hilum of the left lung was occluded by blocking forceps for 45 min. Then, the blocking forceps were removed to continue the blood supply and ventilation (lung vasomotor activity was set as the standard of reperfusion). Finally, rats were killed 120 min after reperfusion. In the IPO group, the hilum of the left lung was blocked for 45 min and temporary reperfusion permitted for 30 s, followed by ischemia for 30 s, for three cycles [12]. Finally, reperfusion was carried out for 120 min, and the remainder of the procedure was identical to that for the I/R group. In the Levo group, 2ml of levosimendan (0.1 µmol/kg; OrionPharma, Espoo, Finland) was administered slowly and infused for 10min *via* the femoral vein before blocking forceps were removed, and the remainder of the procedure was identical to that for the I/R group. In the Levo+5-HD group, 15 min before blocking forceps were removed, 5-HD (10 mg/kg; Sigma–Aldrich, St Louis, MO, USA) was administered *via* the femoral vein and then levosimendan (0.1 µmol/kg) given *via* the femoral vein before opening, and the remainder of the procedure was identical to that for the I/R group.

Anesthesia was induced with 10% chloral hydrate (0.3 mL/100 g body weight, i.p.). An animal respirator was connected to a tracheal cannula after tracheotomy to control respiratory rhythm: breathing rate, 70/min; inspiratory/expiratory time: 1:2; tidal volume: 12 ml/kg. The thoracic cavity was opened *via* the fifth intercostal of the left anterior chest. The fourth and fifth ribs were cut for exposure. Physiological (0.9%) saline was injected *via* the femoral vein to dilute heparin to 100 U/kg. Five minutes’ later, the hilum of the left lung was blocked from the left upper lung to the hilum of the lung at the end of expiration (no vasomotor was set as the standard of blockage). Simultaneously, the tidal volume was adjusted to 2/3 of its original level. After 45 min, the blocking forceps were removed, the tidal volume changed to its original level, and reperfusion carried out for 120 min.

### Collection and handling of samples

Rats were killed by incision of the hilum of the left lung when the experiment was complete. Left-lung samples were placed in physiological saline on ice to clean-off blood stains and dried using filter paper. Upper-lung tissue was placed into 4% PARAformaldehyde solution for immobilization for 24 h. After immobilization, the pathological morphology of lung tissue was observed through routine embedding with paraffin wax, slicing, and hematoxylin & eosin (H&E) staining. Middle-lung tissue was used to determine the apoptosis index (AI) of lung tissue cells and expression of active caspase-3, Bax and Bcl-2. Lower-lung tissue (1 × 1 × 1 mm) was placed in 2.5% glutaraldehyde solution for immobilization. Observation was carried out with an electron microscope after slicing.

### Determination of apoptosis in lung tissue cells using the terminal deoxynucleotidyl transferase dUTP nick end labeling (TUNEL) assay

The TUNEL assay was employed with an Apoptosis Detection kit (Roche, Basel, Switzerland) according to manufacturer instructions. Apoptotic cells contained brown-yellow granules in the cytoplasm. The number of apoptotic cells in five high-power fields at random (×400 magnification) was calculated. The AI was expressed as the number of apoptotic cells/100 cells (%).

### Determination of expression of active caspase-3, Bax and Bcl-2 proteins by immunohistochemical staining and western blotting

Paraffin wax slices were created and then dewaxed using dimethylbenzene. Ethanol hydration was carried out and rinsing with phosphate-buffered saline (PBS; twice every 5 min) completed. Incubation with 0.5% periodic acid was carried out for 15 min to inactivate endogenous enzymes. Rinsing with PBS twice every 5 min was completed. Microwave repairing of antigen lasted for 20 min. Rinsing with PBS thrice every 5 min was completed. Normal serum (50 μL), which was the same that used for the enzyme-labeled antibody, was added, and allowed to react at room temperature for 10 min. The liquid supernatant of tissue was extracted and 50 μL primary antibody added, followed by incubation at 4°C overnight. Rinsing with PBS thrice every 5 min was completed. Biotin-labeled secondary antibody (50 μl) was added followed by incubation at 37°C for 40 min. Rinsing with PBS thrice every 5 min was completed. 3,3’-Diaminobenzidine was added for 10 min. Rinsing with running water and counterstaining with hematin was done. Hyalinization and sealing with dimethylbenzene was then carried out. Observation under a light microscope (Motic, Richmond, BC, Canada) was focused on searching for brown-yellow granules in the cytoplasm. An Advanced 310 Image Processing system (Motic) was connected to the microscope and images taken.

Equal amounts of tissues lysates were separated by SDS polyacrylamide gel electrophoresis. The separated proteins were electrotransferred to the PVDF membrane. The membrane containing the proteins was used for immunoblotting with required antibodies. The protein bands were scanned and quantified as a ratio to β-actin control.

### Measurement of CSE activity

The CSE activity was measured with methylene blue method. CSE can catalyze L- cysteine to produce H2S with the coenzyme pyridoxal 5 ‘- phosphate, so CSE activity can be expressed as the generation rate of H2S in unit tissue indirectly.

### Statistical analyses

Data are expressed as $\bar{x}$±s. One-way ANOVA was used for comparison among groups. SPSS v13.0 (SPSS, Chicago, IL, USA) was used for analyses.

## Results

### Apoptosis of lung tissue cells

**Figure 1:**
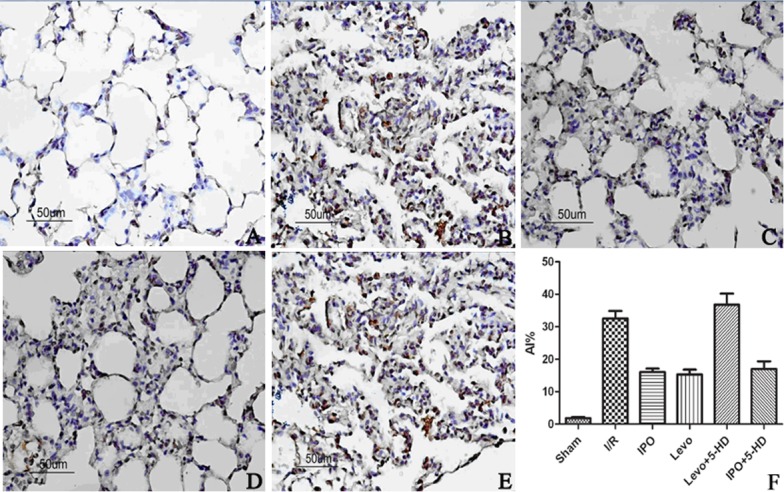
TUNEL staining of apoptotic lung tissue cells in the five groups (×400 magnification). A: Sham group; B: I/R group; C: IPO group; D: Levo group; E: Levo+5-HD group. Apoptotic cells are stained brown-yellow.

Compared with the sham group, the AI in I/R and Levo+5-HD groups was significantly higher (P<0.05), but significant differences between I/R and Levo+5-HD groups were not observed. Compared with I/R and Levo+5-HD groups, the AI in IPO and Levo groups was significantly lower (P<0.05), but significant differences between IPO and Levo groups were not observed. Apoptotic cells were mainly alveolar epithelial and vascular endothelial cells (Fig. 1 and Table 1).

**Table 1:** The AI, expression of active caspase-3, Bax and Bcl-2, and comparison of the ratio between Bcl-2 and Bax in lung tissue (n = 10, $\bar{x}$±s).

**Group**	**AI(%)**	**Active caspase-3**	**Bax**	**Bcl-2**	**Bcl-2/Bax**
Sham	1.87±0.37	0.44±0.04	0.11± 0.01	0.16±0.02	1.45±0.10
I/R	32.42±4.57*	0.83±0.07*	0.23± 0.02*	0.28±0.03*	1.20±0.16*
IPO	15.43±2.32* ^#^	0.69±0.05* ^#^	0.18± 0.02* ^#^	0.33±0.02* ^#^	1.84±0.33* ^#^
DE	15.19±3.46* ^#^	0.65±0.04* ^#^	0.17± 0.03* ^#^	0.34±0.03* ^#^	1.86±0.27* ^#^
DE+5-HD	31.51±5.33*	0.81±0.07*	0.22± 0.03*	0.29±0.04*	1.22±0.15*

Compared with Sham group: *P<0.05;

Compared with I/R group: ^#^P<0.05

### Expression of active caspase-3, Bax and Bcl-2 proteins

**Figure 2:**
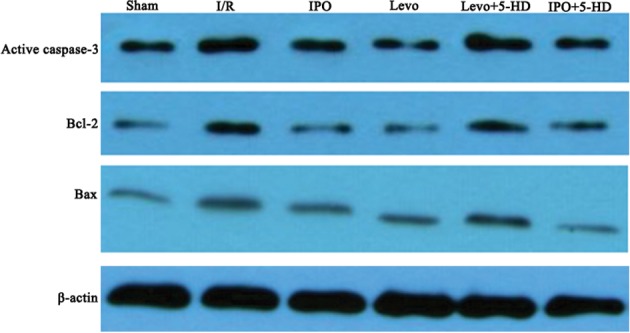
Western blotting results of active caspase-3, Bax and Bcl-2 in different groups.

Compared with the sham group, expression of active caspase-3, Bax and Bcl-2 proteins in I/R and Levo+5-HD groups increased significantly (P<0.05), the ratio between expression of Bcl-2 and Bax decreased significantly (P<0.05), and there were no significant differences between I/R and Levo+5-HD groups. Compared with I/R and Levo+5-HD groups, expression of active caspase-3 and Bax proteins in IPO and Levo groups was significantly lower (P<0.05), whereas expression of Bcl-2 protein as well as the ratio between Bcl-2 and Bax were significantly higher (P<0.05), and there were no significant differences between IPO and Levo groups (Table 1). The results of Western blotting were shown in Fig. 2.

### Morphology

**Figure 3:**
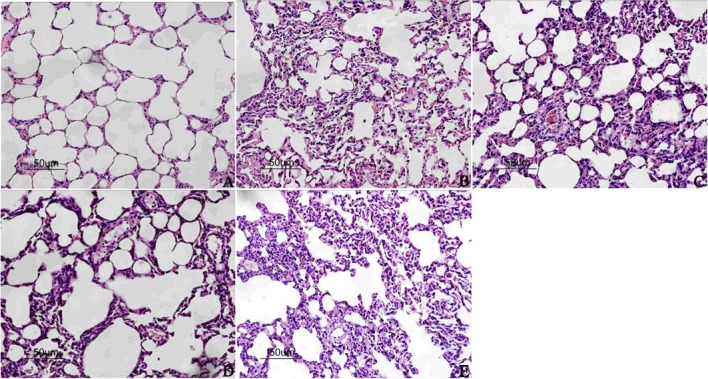
H&E staining of lung tissue (×200 magnification). A: Sham group; B: I/R group; C: IPO group; D: Levo group; E: Levo+5-HD group.

The structure of pulmonary interstitial tissue and the alveolus in the sham group was intact and clearly visible under light microscopy without exudation into the alveolar space or infiltration of inflammatory cells. In I/R and Levo+5-HD groups, obvious pathological morphology was observed: atelectasis complicated with pulmonary emphysema; interstitial edema; infiltration of inflammatory cells; erythrocyte exudation in the alveolar space). However, in IPO and Levo groups, less interstitial edema and infiltration of inflammatory cells, as well as a significant decrease in bleeding and exudation, were noted, so the severity of tissue injury was lower than that in the I/R group (Fig. 3).

**Figure 4:**
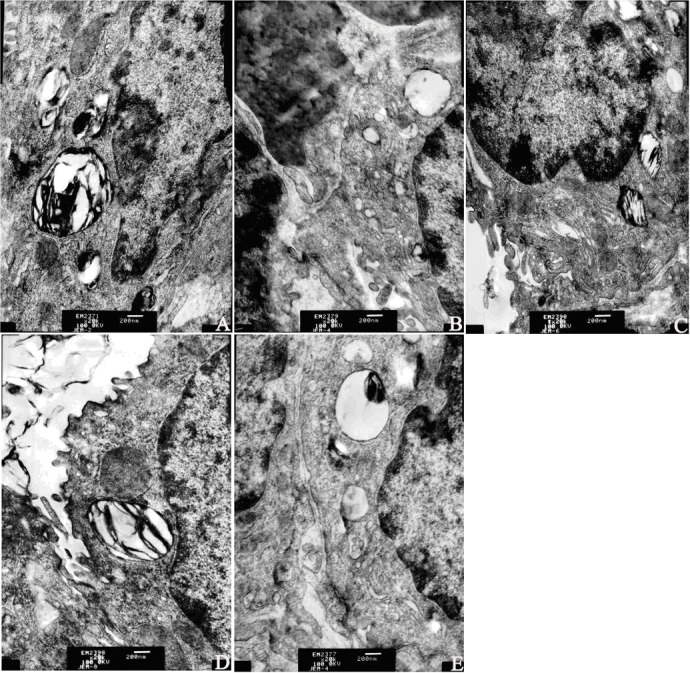
Ultrastructure of type-II alveolar epithelial cells in lung tissue (×20,000 magnification). A: Sham group; B: I/R group; C: IPO group; D: Levo group; E: Levo+5-HD group.

Under electron microscopy, the structure of type-II alveolar epithelial cells in the sham group was intact, and mitochondrial cristae were clear without changes in the lamellar body or loss of microvilli. The oval-shaped karyon was also visible. In I/R and Levo+5-HD groups, the mitochondria of type-II alveolar epithelial cells were swollen and increased in number. Also, the electron density of mitochondrial matrices decreased, and disordered structures of cristae, obvious evacuation of the lamellar body, and lose of microvilli were noted. Some karyons were found to be polymorphic. In IPO and Levo groups, the mitochondria of type-II alveolar epithelial cells were slightly swollen and had less disordered cristae, mild evacuation of the lamellar body and loss of microvilli also noted, and most karyons were oval (Fig. 4).

### CSE activity

**Figure 5:**
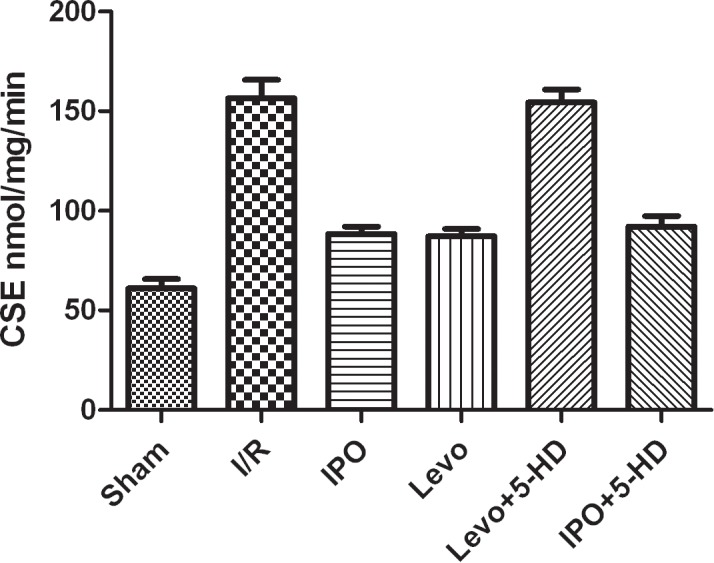
Results of CSE activity.

The results of CSE activity was shown in Fig. 5. It suggested that 5-HD blocked LEVO and did not block IPO.

## Discussion

The protective function of IPO postconditioning against the injury caused by reperfusion has been confirmed in animal and humans experiments, but its clinical application is limited because of medical ethics and potential risks. Drug postconditioning is a further stage of IPO postconditioning: after ischemia, drugs are used before reperfusion (or within minutes after the beginning of reperfusion) [18] to alleviate the injury caused by reperfusion. Drug postconditioning is non-invasive and convenient.

Levosimendan is used widely to treat heart failure [19, 20]. A possible mechanism of action is enhancement of myocardial contraction by increasing the sensitivity between Ca^2+^ and myofilaments, leading to depletion of [Ca^2+^]_i_. Simultaneously, levosimendan can activate the K_ATP_ channels of vascular smooth muscle cells and myocardial cells and inhibit the internal flow of Ca^2+^. It can also meanwhile, activate sodium–calcium exchangers for external flow of Ca^2+^ so as to decrease [Ca^2+^]_i_, dilate vessels and relieve the preload and afterload of the heart [13–16, 21]. Furthermore, levosimendan is also an opener of the K^+^ channels of the plasma membrane and mitochondrial membranes. Hence, it can protect the myocardium by activating K^+^ channels and inhibiting mitochondrial apoptosis [22].

We observed few apoptotic cells in the sham group but more apoptotic cells in the I/R group. Obvious injury to lung tissue was observed under light and electron microscopy, which suggested that apoptosis played a part in the development of LIRI. For rats that underwent IPO and Levo postconditioning, apoptosis in lung tissue cells decreased significantly and change in the morphology of lung tissue was less significant, which suggested that IPO postconditioning can decrease the degree of LIRI. Lung protection due to Levo postconditioning was identical to that of IPO postconditioning. Interestingly, many apoptotic cells and obvious morphological injury were noted in Levo+5-HD and I/R groups. This was because 5-HD, a specific mitochondrial-sensitive K^+^ channel blocker, inactivated the lung-protective effect induced by Levo postconditioning. This finding demonstrated that Levo postconditioning opposes LIRI by activating the mitoK_ATP_ pathway.

Apoptosis is under the regulation of genes and also an important part of the mechanism of ischemia–reperfusion injury [17]. Bcl-2 and Bax belong to a family of Bcl-2 proteins. If Bcl-2 expression is high, homodimers or heterodimers of Bcl-2/Bcl-2 are created, and both can inhibit apoptosis. If Bax expression is high, homodimers of Bax/Bax are created, which boosts apoptosis. The change in the ratio of expression of these two factors determines if apoptosis occurs within the target location as well as the severity of apoptosis [23]. Caspases are cytokines that take part in the regulation of apoptosis, and are central actors in activation of apoptotic cascades. Active caspase-3 concentration is closely related to the rate of apoptosis [24]. We showed that expression of Bcl-2, Bax and active caspase-3 proteins in lung tissue in I/R and Levo+5-HD groups was upregulated significantly, and that the Bcl-2/Bax ratio decreased significantly. Also, expression of Bax and active caspase-3 proteins in lung tissue in IPO and Levo groups decreased significantly, whereas expression of Bcl-2 protein and the Bcl-2/Bax ratio was up-regulated significantly. These results suggested that the apoptotic mechanism of levosimendan against LIRI could include upregulation of expression of Bcl-2 protein, downregulation of expression of Bax protein, higher Bcl-2/Bax ratio, and lower dependence upon active caspase-3.

In LIRI, the main mechanism of apoptosis involves free-radical damage and Ca^2+^ overload. It has been confirmed that IPO postconditioning can relieve ischemia–reperfusion by opening mitoK_ATP_ channels [25]. The present study suggested that levosimendan, as a type of drug postconditioning, alleviated LIRI by activating mitoK_ATP_ channels, and that this protective effect was blocked by the specific mitoK_ATP_ channel blocker 5-HD. However, 5-HD did not block IPO. Opening mitoK_ATP_ channels depolarizes the inner membranes of mitochondria, thereby promoting internal flow of K^+^ and relieving Ca^2+^ overload within mitochondria. These actions protect the activity of mitochondrial enzymes and increase the volume of mitochondria matrices. Increasing the volume of mitochondrial matrices increases ATP synthesis and activates the mitochondrial respiratory chain to decrease production of oxygen free radicals. Activation of mitoK_ATP_ channels also inhibits release of cytochrome C from mitochondria to resist apoptosis by maintaining the mitochondrial permeability transition pore (MPTP) [26].

## Conclusions

Levosimendan postconditioning can protect against LIRI. This protective effect includes activation of mitoK_ATP_ channels, relief of Ca^2+^ overload, upregulation of the Bcl-2/Bax ratio, and reduction of active caspase-3 expression.
